# Improved Output Power of GaN-based VCSEL with Band-Engineered Electron Blocking Layer

**DOI:** 10.3390/mi10100694

**Published:** 2019-10-12

**Authors:** Huiwen Luo, Junze Li, Mo Li

**Affiliations:** 1 Institute of Electronic Engineering, China Academy of Engineering Physics, Mianyang 621999, China; luohuiwen@mtrc.ac.cn; 2 Microsystem and Terahertz Research Center, China Academy of Engineering Physics, Chengdu 610200, China

**Keywords:** GaN-based vertical-cavity surface-emitting laser (VCSEL), composition-graded Al_x_Ga_1−x_N electron blocking layer (EBL), electron leakage

## Abstract

The vertical-cavity surface-emitting laser (VCSEL) has unique advantages over the conventional edge-emitting laser and has recently attracted a lot of attention. However, the output power of GaN-based VCSEL is still low due to the large electron leakage caused by the built-in polarization at the heterointerface within the device. In this paper, in order to improve the output power, a new structure of p-type composition-graded Al_x_Ga_1−x_N electron blocking layer (EBL) is proposed in the VCSEL, by replacing the last quantum barrier (LQB) and EBL in the conventional structure. The simulation results show that the proposed EBL in the VCSEL suppresses the leaking electrons remarkably and contributes to a 70.6% increase of the output power, compared with the conventional GaN-based VCSEL.

## 1. Introduction

Vertical-cavity surface-emitting lasers (VCSELs) exhibit several advantages over edge-emitting lasers (LDs), including high-speed direct modulation, circular mode profile, low threshold current, etc. [1,2,3]. Recently, although GaAs-based VCSELs that emit red or infrared light have been commercialized and applied to various products, it is still hoped that VCSELs will not only cover the red area, but also the blue and green spectrum region, thus constituting the ternary color of light for illumination, display and communication of the next generation [4]. As wide-bandgap materials, the bandgap width of gallium nitride (GaN) and its alloy materials are continuously adjustable from 0.7 eV to 6.2 eV, covering near-red, green, blue, and ultraviolet. They have become the main material for manufacturing short-wavelength light-emitting diodes (LEDs) and LDs. Recently, the GaN-based materials have been applied to VCSELs and achieved important progress [5]. Universities and research institutes such as National Chiao-Tung University [6], Nichia Corporation [7], University of California, Santa Barbara [8], Sony Corporation [1], Xiamen University [9] and Meijo University [10] conducted good work on the designing and manufacturing of blue GaN-based VCSELs. In 2018, Stanley Electric Co., Ltd. demonstrated a GaN-based VCSEL with an output power of 7.6 mW by reducing both the internal loss and the reflectivity of the front cavity mirror [11]. This structure achieved the highest output power of a GaN-based VCSEL ever produced to the best of our knowledge. However, it is still relatively low as a result of ignoring other mechanisms. For example, for InP and other semiconductor-based lasers, lower threshold and higher output power can be achieved by designing the micro-cavities, which may also have good effects in GaN-based VCSELs [12,13,14].

Beyond designing the micro-cavities, in order to obtain higher output power, methods which maximize the radiative recombination rate have been adopted by minimizing the electron leakage out of the active region. Among those methods, Al_x_Ga_1−x_N electron blocking layer (EBL) is usually placed between the active region and p-GaN layer, since it provides a higher energy barrier in the conduction band between quantum wells (QWs) and the p-layers. The higher energy barrier in the conduction band contributes to the confinement of the electrons in the active region, so stronger output power can be achieved [15]. However, the very commonly used Al_x_Ga_1−x_N EBL sometimes cannot efficiently reduce electron leakage due to the polarization charge in that layer, which decreases the barrier height for electron transport [16,17]. As such, the blocking effects of Al_x_Ga_1−x_N EBL on electron overflow are limited [18]. In addition, there are big differences in the polarization value and the energy barrier height in the conduction band between Al_x_Ga_1−x_N EBL and In_x_Ga_1−x_N last quantum barrier (LQB), which results in a large built-in polarization field and significant free electron accumulation at the heterointerface between the two layers. This free electron accumulation outside of the active region severely degrades the internal quantum efficiency. To solve this problem, Zhang et al. proposed a design which replaces the In_x_Ga_1−x_N LQB with a tapered Al_x_Ga_1−x_N LQB in GaN-based LD [19], and Lin et al. proposed another structure which replaces the Al_x_Ga_1−x_N EBL with a composition-graded Al_x_Ga_1−x_N EBL in the VCSEL [20].

In this work, our concept is to improve the output power by optimizing the carrier transport. We proposed an improved GaN-based VCSEL structure called GVCSEL to reduce the electron leakage. In GVCSEL, the LQB and EBL in the conventional GaN-based VCSEL are replaced with a new layer which consists of a composition-graded p-Al_x_Ga_1−x_N layer and a p-Al_x_Ga_1−x_N layer. The physical and optical properties of the GVCSEL are investigated numerically with the Photonic Integrated Circuit Simulator in 3D (PICS3D) software (Crosslight Corporation, Vancouver, BC, Canada). The results show that the GVCSEL effectively confines the electrons in the active region and has a more uniform carrier distribution. This contributes to a better radiative recombination and helps achieve a 70.6% increase of the output power compared with the conventional GaN-based VCSEL.

## 2. Device Structure and Simulation Parameters

**Figure 1:**
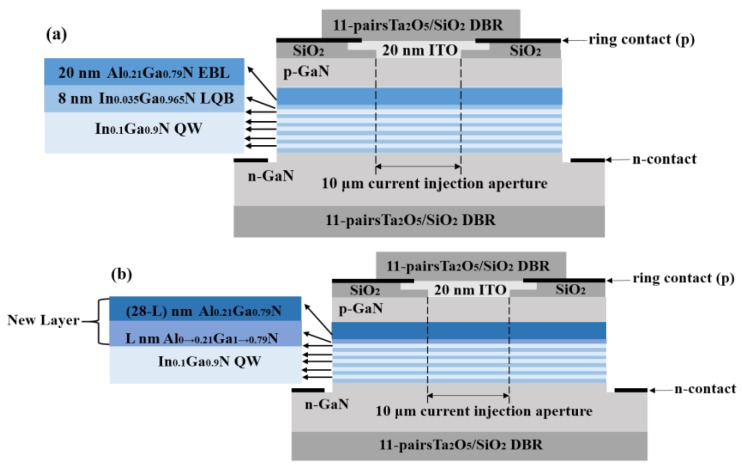
Schematic diagrams of the (**a**) conventional gallium nitride (GaN)-based vertical-cavity surface-emitting laser VCSEL (CVCSEL) and (**b**) proposed structure which is formed by replacing the LQB and EBL in the CVCSEL with a new layer (GVCSEL).

In this work, the conventional GaN-based VCSEL was used for comparison, as shown in Figure 1a [21], which consists of 11 pairs of Ta_2_O_5_/SiO_2_ as the bottom and top distributed Bragg reflectors (DBRs), respectively. Then, there is a 5.3 μm-thick n-type GaN layer (n-doping = 2.5 × 10^18^ cm^−3^) and five periods of In_0.1_Ga_0.9_N/In_0.035_Ga_0.965_N multi-quantum wells (MWQs). The thicknesses of the well and the barrier are 4 nm and 8 nm, respectively, the n-doping of the barrier is 1 × 10^18^ cm^−3^, whilst the LQB is undoped. Next there is a 20 nm-thick Al_0.21_Ga_0.79_N (p-doping = 5 × 10^18^ cm^−3^) EBL, followed by a 0.54 μm-thick p-type GaN layer (p-doping = 1 × 10^18^ cm^−3^). On the p-type GaN layer, a 20 nm-thick SiO_2_ is employed as the current-confined layer. Following that, a 20 nm-thick indium-tin-oxide (ITO) layer (p-doping = 1 × 10^19^ cm^−3^) is employed as the current spreading layer and the diameter of the current injection aperture is designed to be 10 μm. The top metal ring contact confining the optical mode is 12 μm in diameter. Figure 1b shows the schematic diagram of the GVCSEL in this study, which is formed by replacing the 8 nm-thick In_0.035_Ga_0.965_N LQB and 20 nm-thick Al_0.21_Ga_0.79_N EBL in the conventional GaN-based VCSEL with a new layer. The new layer consists of a p-Al_0→0.21_Ga_1→0.79_N (p-doping = 5 × 10^18^ cm^−3^) layer and a p-Al_0.21_Ga_0.79_N (p-doping = 5 × 10^18^ cm^−3^) layer, where the thickness of the first layer is L nm and the thickness of the second layer is (28-L) nm. The GaN-based VCSEL samples with L equals 6 nm, 8 nm and 16 nm are named GVCSEL1, GVCSEL2 and GVCSEL3, respectively, and their parameters are given in Table 1. The conventional GaN-based VCSEL is named CVCSEL for comparison.

The physical features and optical properties of the GVCSEL and CVCSEL were investigated numerically with the Simupics3d program of Crosslight software. An important issue in simulation is the selection of proper parameters in the physical models. In this study, we used a Mg activation energy of 170 meV for GaN, which was assumed to increase by 3 meV per Al% for AlGaN [12]. The Shockley‒Read‒Hall (SRH) lifetime and the Auger recombination coefficient were estimated to be 100 ns and 1 × 10^−34^ cm^6^s^−1^, respectively [22]. The built-in polarization caused by piezoelectric polarization and spontaneous polarization was represented by fixed interface charges at every heterointerface within the device, which was calculated using the methods developed by Fiorentini et al. [23]. Here we took 50% of the theoretical value by setting the polarization screening to 0.5 [24]. We also considered the energy band offset ratio as 50:50 [25]. Other material parameters in the simulation can be found in [26].

## 3. Results and Discussions

**Figure 2:**
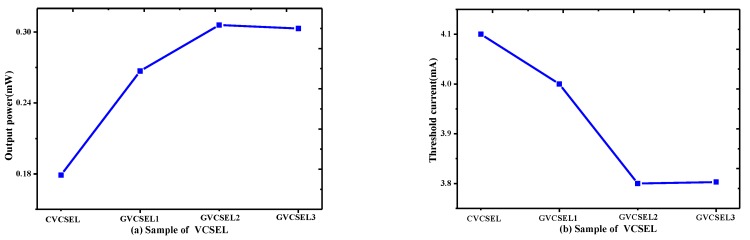
(**a**) Output power at the injection current of 6 mA and (**b**) threshold current of CVCSEL and GVCSEL.

Since the output power and the threshold current are interlinked, investigating both of them is crucial to understand the performance of GaN-based VCSELs [17], we calculated the two performances of CVCSEL and GVCSEL1-GVCSEL3, as shown in Figure 2. It was found that the output power of CVCSEL was 0.179 mW and was lower than that of the GVCSELs in Figure 2a. In addition, the output power increased from 0.267 mW to 0.306 mW when the thickness of L of the p-Al_0→0.21_Ga_1→0.79_N in the GVCSEL was increased from 6 nm to 8 nm, while with further increase of L from 8 nm to 16 nm, the output power decreased from 0.306 mW to 0.303 mW. Thus, the highest output power was obtained in GVCSEL2, which achieved a 70.6% increase of output power compared with the CVCSEL. Figure 2b shows that the current threshold of the four samples first decreased and then went up. The lowest threshold current was also achieved in GVCSEL2.

**Figure 3:**
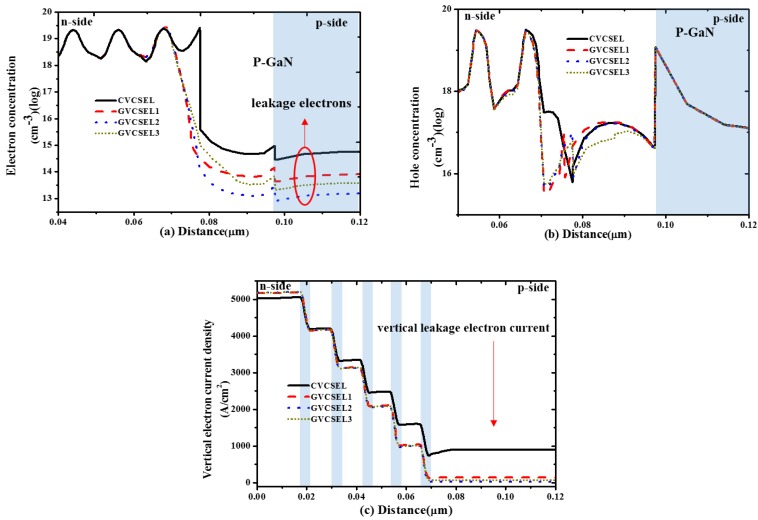
The distribution of (**a**) electron concentration; (**b**) hole concentration; (**c**) vertical electron current density, of GVCSEL1-3 and CVCSEL at an injection current of 6 mA.

**Figure 4:**
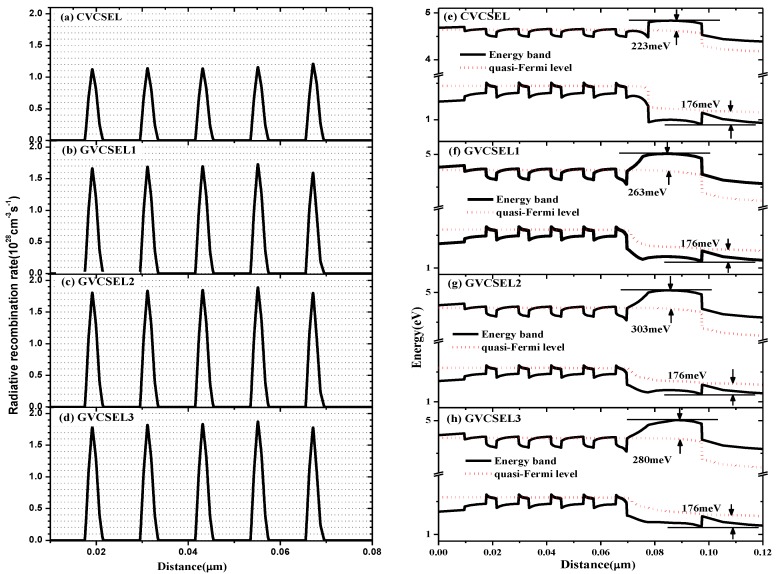
Distribution of (**a**–**d**) the radiative recombination rate and (**e**–**h**) the energy band of GVCSELs and CVCSEL at the injection current of 6 mA.

In order to reveal the origin of the observations in Figure 2, we calculated the distribution of the electrons and the holes and the current density flowing along the vertical direction near the active region of the four samples at an injection current of 6 mA, as shown in Figure 3. We also calculated the distribution of the radiative recombination rate as shown in Figure 4a–d. In Figure 3a, the highest electron concentration in the p-GaN layer was obtained in CVESEL, thus there was the largest electron leakage. For the GVCSELs, the electron concentration in the p-GaN layer decreased when L was increased from 6 nm to 8 nm. However, the electron concentration in the p-GaN layer increased when the thickness of L went up from 8 nm to 16 nm. Therefore, the lowest electron concentration in the p-GaN layer was obtained in GVCSEL2, which means that the largest reduction of the electron leakage was obtained in GVCSEL2. Thus, the reduction of electron leakage is one of the origins of the increase of output power and the reduction of the threshold current for CVCSEL-GVCSEL3. Figure 3b illustrates that the hole concentration in p-GaN layers of the GVCSELs was almost the same with that of the CVCSEL, which means that the hole injection is not changed in GVCSELs compared to CVCSEL. Thus, the hole injection is not the origin of the change of output power and threshold current for the samples. Figure 3c demonstrates that the electron overflow of GVCSEL was efficiently suppressed and the most effective suppression of the electron overflow was obtained in GVCSEL2. There was more uniform carrier distribution with the reduction of electron overflow. As a result, the radiative recombination rate in GVCSEL was improved, as can be seen in Figure 4a–d, and the highest one was obtained in GVCSEL2, as shown in Figure 4c. In summary, Figure 3 and Figure 4a–d show that the increase of the output power and the decrease of the threshold current for samples of GaN-based VCSEL can be attributed to the reduced electron leakage and an increased radiative recombination rate.

Finally, for the purpose of investigating in-depth the origin of the reduced leaking electrons in the GVCSEL, we calculated the distribution of the energy band at an injection current of 6 mA, as plotted in Figure 4e–h. In CVCSEL, the effective barrier height of the EBL for the electrons was 223 meV, which was lower than that of GVCSEL. Therefore, in CVCSEL the electrons were easier to leak into the p-GaN layer. The increase of the electron leakage resulted in a more nonuniform carrier distribution in the active region, thus lowering the radiative recombination rate and the output power. For GVCSELs, when the thickness of L was increased from 6 nm to 8 nm, the effective barrier height increased from 263 meV to 303 meV. But the effective barrier height decreased from 303 meV to 280 meV when the thickness of L further increased from 8 nm to 16 nm. This means that the highest effective barrier height was the origin of the highest output power obtained in GVCSEL2. We can also see that the effective barrier height of EBL for the holes was about 176 meV in CVCSEL and all three GVCSELs. Therefore, the ability inject holes into the active region is almost the same in the CVCSEL and the GVCSELs, as shown in Figure 3b. In summary, the origin of the increased output power for the GVCSEL is not the rate of the injection holes but the suppressed electron leakage.

## 4. Conclusions

In our work, in order to improve the output power of GaN-based VCSEL, we proposed a structure called GVCSEL, based on the concept of modulating the carrier distribution. A new layer was used by combining the LQB and EBL in the conventional GaN-based VCSEL, which was made up of a L nm-thick composition-graded p-Al_x_Ga_1−x_N layer and a (28-L) nm-thick p-Al_0.21_Ga_0.79_N layer and the Al component in the graded Al_x_Ga_1−x_N changed from 0 to 0.21. The thickness of L for the three GVCSEL samples was selected to be 6 nm, 8 nm and 16 nm. The numerical simulation results showed that all the GVCSELs had improved output power. The higher output power in the GVCSELs is an attribute of the decrease of the large built-in polarization field and the reduction of the free electron accumulation at the heterointerface between the LQB and the Al_x_Ga_1−x_N EBL. This further helped the suppression of the electron leakage and contributed to a more uniform carrier distribution in the active region, resulting in a higher radiative recombination rate in the quantum wells. What is more, this proposed layer also introduced a stronger quantum barrier which can confine the carriers in the quantum wells. In the GVCSELs, the one with a thickness of L of 8 nm obtained the highest output power, which was 70.6% stronger over that of the CVCSEL, since it had higher effective barrier height in the conduction band for electrons. Therefore, the GVCSEL output power can be accurately designed by selecting the thickness of L.

## Figures and Tables

**Table 1:** The thickness of p-Al_0.21_Ga_0.79_N layer and p-Al_0→0.21_Ga_1→0.79_N layer of GVCSEL.

Sample Name	GVCSEL1	GVCSEL2	GVCSEL3
p-Al_0.21_Ga_0.79_N	22 nm	20 nm	10 nm
p-Al_0→0.21_Ga_1→0.79_N	6 nm	8 nm	16 nm
